# ASO Author Reflections: Is Vascular Cell Adhesion Molecule-1 (VCAM-1) a Promising Biomarker in Urothelial Carcinoma of the Bladder?

**DOI:** 10.1245/s10434-022-11702-1

**Published:** 2022-04-12

**Authors:** Keiichiro Mori, Victor M. Schuettfort, Shin Egawa, Eva Compérat, Shahrokh F. Shariat

**Affiliations:** 1 Department of Urology, Medical University of Vienna, Vienna, Austria; 2 Department of Urology, The Jikei University School of Medicine, Tokyo, Japan; 3 Department of Urology, University Medical Center Hamburg-Eppendorf, Hamburg, Germany; 4 Department of Pathology, Medical University of Vienna, Vienna, Austria; 5 Institute for Urology and Reproductive Health, Sechenov University, Moscow, Russia; 6 Hourani Center for Applied Scientific Research, Al-Ahliyya Amman University, Amman, Jordan; 7 Department of Urology, Weill Cornell Medical College, New York, NY USA; 8 Department of Urology, University of Texas Southwestern, Dallas, TX USA; 9 Karl Landsteiner Institute of Urology and Andrology, Vienna, Austria; 10 Department of Urology, Second Faculty of Medicine, Charles University, Prague, Czech Republic

## Past

Outcome prediction models for patients with urothelial carcinoma of the bladder (UCB) are less readily available for clinical use, primarily because of their inability to capture the complete potential of host–tumor interactions.^1^ Thus, identifying preoperative biomarkers capable of capturing the biological and clinical potential of each tumor variety is crucial for further refinement of risk stratification in patients with UCB.^2,3^ Vascular cell adhesion molecule-1 (VCAM-1) involved in cellular adhesion is closely associated with inflammation, immunological disorders, tumor angiogenesis, and metastasis.^4^ Although VCAM-1 is reportedly associated with oncological outcomes in several tumor varieties,^4^ the predictive and/or prognostic value of blood VCAM-1 remains relatively unexplored in patients with UCB undergoing radical cystectomy (RC).

## Present

This consecutive cohort study enrolled 1036 patients with clinically nonmetastatic advanced UCB having undergone RC to investigate the relationship between preoperative plasma VCAM-1 levels and the established features of UCB invasion, metastasis, and survival outcomes.^5^ Beyond multivariable modeling, we used predictive accuracy testing and decision curve analysis (DCA) to assess the value of preoperative VCAM-1 as a biomarker in real-world clinical settings. The results indicate that elevated preoperative VCAM-1 levels were not only associated with worse oncological outcomes but also predicted biologically and clinically aggressive disease. In DCA, the reference models for lymph node metastasis, non-organ-confined disease, recurrence-free survival, and cancer-specific survival prediction resulted in significant improvements after addition of VCAM-1.

## Future

This study demonstrates that addition of VCAM-1 improved the discriminatory power of predictive/prognostic models significantly. Therefore, VCAM-1 might be a valuable biomarker in determining the need for perioperative chemotherapy and the extent of lymphadenectomy required. VCAM-1 is associated with ease of procurement, low cost, high sample homogeneity, and potential to improve early outcome prediction and prognosis. However, further study is warranted for VCAM-1 to be determined as a candidate biomarker worth integrating into prospective clinical trials to enhance currently available tools used for risk stratification in patients with UCB, and assess its value in the era of immunotherapy.

## References

[1] Karakiewicz PI, Shariat SF, Palapattu GS, et al. Nomogram for predicting disease recurrence after radical cystectomy for transitional cell carcinoma of the bladder. *J Urol.* 2006;176(4 Pt 1):1354-1361; discussion 1361–1352. 10.1016/j.juro.2006.06.025 16952631

[2] Mori K, Schuettfort VM, Katayama S, et al. The value of preoperative plasma VEGF levels in urothelial carcinoma of the bladder treated with radical cystectomy. *Eur Urol Focus.* 2021. 10.1016/j.euf.2021.08.006 34454852

[3] Mori K, Miura N, Mostafaei H (2020). Prognostic value of preoperative hematologic biomarkers in urothelial carcinoma of the bladder treated with radical cystectomy: a systematic review and meta-analysis. Int J Clin Oncol..

[4] Kong DH, Kim YK, Kim MR, Jang JH, Lee S. Emerging roles of vascular cell adhesion molecule-1 (VCAM-1) in immunological disorders and cancer. *Int J Mol Sci.* 2018;19(4). 10.3390/ijms19041057 PMC5979609 29614819

[5] Mori K, Schuettfort VM, Katayama S, Laukhtina E, Pradere B, Quhal F, Sari Motlagh R, Mostafaei H, Grossmann NC, Rajwa P, Teoh JYC, Resch I, Fajkovic H, Moschini M, D’andrea D, Abufaraj M, Karakiewicz PI, Lotan Y, Scherr D, Egawa S, Compérat E, Shariat SF. Prognostic role of preoperative vascular cell adhesion molecule-1 plasma levels in urothelial carcinoma of the bladder treated with radical cystectomy. *Ann Surg Oncol.* 2022. 10.1245/s10434-022-11575-4 10.1245/s10434-022-11575-4 PMC9246812 35347517

